# The usefulness of twenty-four molecular markers in predicting treatment outcome with combination therapy of amodiaquine plus sulphadoxine-pyrimethamine against falciparum malaria in Papua New Guinea

**DOI:** 10.1186/1475-2875-7-61

**Published:** 2008-04-19

**Authors:** Jutta Marfurt, Ivo Müller, Albert Sie, Olive Oa, John C Reeder, Thomas A Smith, Hans-Peter Beck, Blaise Genton

**Affiliations:** 1 Swiss Tropical Institute, Socinstrasse 57, P.O. Box, CH-4002 Basel, Switzerland; 2 Papua New Guinea Institute of Medical Research, Goroka, P.O. Box 60, EHP 441, Papua New Guinea; 3 Menzies School of Health Research, P.O. Box 41096, Casuarina, Darwin, NT 0811, Australia; 4 Papua New Guinea Institute of Medical Research, Maprik, P.O. Box 400, ESP 533, Papua New Guinea; 5 International Health Research Strategy, Burnet Institute for Medical Research and Public Health, P.O. Box 2284, Melbourne VIC 2001, Australia; 6 Swiss Tropical Institute, Department of Public Health and Epidemiology, Socinstrasse 57, P.O. Box, CH-4002 Basel, Switzerland; 7 Swiss Tropical Institute, Department of Medical Parasitology and Infection Biology, Socinstrasse 57, P.O. Box, CH-4002 Basel, Switzerland; 8 Ifakara Health Research & Development Centre, P.O. Box 78373, Dar es Salaam, Tanzania

## Abstract

**Background:**

In Papua New Guinea (PNG), combination therapy with amodiaquine (AQ) or chloroquine (CQ) plus sulphadoxine-pyrimethamine (SP) was introduced as first-line treatment against uncomplicated malaria in 2000.

**Methods:**

We assessed *in vivo *treatment failure rates with AQ+SP in two different areas in PNG and twenty-four molecular drug resistance markers of *Plasmodium falciparum *were characterized in pre-treatment samples. The aim of the study was to investigate the association between infecting genotype and treatment response in order to identify useful predictors of treatment failure with AQ+SP.

**Results:**

In 2004, Day-28 treatment failure rates for AQ+SP were 29% in the Karimui and 19% in the South Wosera area, respectively. The strongest independent predictors for treatment failure with AQ+SP were *pfmdr1 *N86Y (OR = 7.87, *p *< 0.01) and *pfdhps *A437G (OR = 3.44, *p *< 0.01). Mutations found in CQ/AQ related markers *pfcrt *K76T, A220S, N326D, and I356L did not help to increase the predictive value, the most likely reason being that these mutations reached almost fixed levels. Though mutations in SP related markers *pfdhfr *S108N and C59R were not associated with treatment failure, they increased the predictive value of *pfdhps *A437G. The difference in treatment failure rate in the two sites was reflected in the corresponding genetic profile of the parasite populations, with significant differences seen in the allele frequencies of mutant *pfmdr1 *N86Y, *pfmdr1 *Y184F, *pfcrt *A220S, and *pfdhps *A437G.

**Conclusion:**

The study provides evidence for high levels of resistance to the combination regimen of AQ+SP in PNG and indicates which of the many molecular markers analysed are useful for the monitoring of parasite resistance to combinations with AQ+SP.

## Background

The effectiveness of the most widely used first-line antimalarials chloroquine (CQ) and sulphadoxine-pyrimethamine (SP) has been heavily compromised by the emergence and spread of *Plasmodium falciparum *resistance to these drugs. In order to improve treatment efficacy and to delay the development and spread of drug resistance, there is strong advocacy for combination therapy [1]. Though the combination of 4-aminoquinolines or SP with artemisinin derivates is recommended, this option is expensive and several countries have taken an interim step and chose the inexpensive combination of amodiaquine (AQ) or CQ plus SP.

Monitoring of parasite resistance is essential in directing the rational use of antimalarials. Apart from studies assessing *in vivo *drug efficacy and *in vitro *drug sensitivity, molecular markers have been proposed as a means to monitor drug resistant malaria [2]. CQ resistance has been attributed to several mutations occurring in the *P. falciparum *chloroquine resistance transporter gene (*pfcrt*) and *P. falciparum *multidrug resistance gene 1 (*pfmdr1*). Correlation between molecular markers of CQ resistance and *in vivo *treatment outcome has been complex. Whereas several studies have shown the key role of *pfcrt *K76T in conferring *in vivo *resistance to CQ [3,4], the relationship between phenotypic resistance and other *pfcrt *polymorphisms (i.e., C72S/R, M74I/T, N75E/D/K/I, K76T/I/N, I77T, H97Q/L, A144F/T, L148I, L160Y, I194T, A220S, Q271E, N326S/D, I356V/T/L and R371T/I), which have been shown to be associated with CQ resistance *in vitro*, has been little studied in the field. Single-base changes in *pfmdr1 *N86Y, Y184F, S1034C, N1042D and D1246Y have been documented in CQ resistant laboratory strains, but a straightforward association of these polymorphisms with *in vivo *CQ resistance has been questioned by several authors [5,6].

The accumulation of point mutations in *P. falciparum *dihydrofolate reductase (*pfdhfr*) and dihydropteroate synthase (*pfdhps*), two enzymes in the parasite's folate synthesis pathway, is associated with resistance to SP. Though the relationship between polymorphisms in these genes and resistance to SP has been shown *in vitro*, the correlation of different genotypes and clinical treatment outcome varies between different epidemiological settings. Whereas the triple mutation S108N+C59R+N51I in *pfdhfr *has been found to be a good molecular marker for SP resistance by some authors [7-9], others did not confirm the usefulness of this combination of mutations [10,11]. The quintuple mutation *pfdhfr *S108N+C59R+N51I plus *pfdhps *A437G+K540E has been proposed as a useful indicator for monitoring SP resistance in Africa [12], in the Amazon region, the quintuple mutation *pfdhfr *S108N+N51I+I164L plus *pfdhps *A437G+K540E has been shown to be more useful [13]. More recently, several authors have found the double mutation *pfdhfr *C59R plus *pfdhps *K540E to be sufficient to predict treatment failure *in vivo *[14-16]. The most likely reason for these conflicting reports is the fact that, apart from the infecting genotype, response to drug treatment is affected by many factors, such as host immunity, which is related to transmission intensity, and history of drug use in a given area [10,17,18]. As a consequence, the patterns as well as the predictive values of molecular drug resistance markers may vary between different geographical regions. Another problem is that most of the studies looked at only few markers, which does not allow comparing the respective value of each to monitor parasite resistance to specific drugs.

After a long history of 4-aminoquinoline use which has been accompanied by accumulating reports about increasing levels of AQ and CQ resistance [19,20], official drug policy for uncomplicated malaria in Papua New Guinea (PNG) was changed to the combination therapy of AQ or CQ plus SP in 2000. Although high levels of polymorphisms in CQ relevant genes *pfcrt *and *pfmdr1*, and also to a lesser extent in key markers responsible for resistance to SP, have already been reported in PNG [21,22], their association with *in vivo *treatment outcome has never been evaluated.

In this study, the genetic profile of parasites collected from pre-treatment samples of malaria patients attending two health facilities in PNG with known clinical and parasitological outcomes after treatment with AQ+SP was analyzed. Twenty-four key markers in *pfmdr1*, *pfcrt, pfdhfr *and *pfdhps *were determined using a new DNA microarray-based technology. The association between parasite genetic output and treatment response was investigated to identify the most useful predictors of failure with the current first-line regimen in the country.

## Materials and methods

### *In vivo *assessment of drug efficacy

Drug efficacy studies were conducted according to the standardized WHO protocol for low to moderate transmission areas [2] and are described in detail elsewhere [23]. Children between six months and seven years of age were enrolled if they were presenting at the health centre with clinically overt and microscopically confirmed *P. falciparum *malaria and no danger signs for severe or complicated malaria or signs of any other disease, malnutrition or anaemia. Standard AQ plus SP first line-treatment (10 mg AQ per kg on Day 0, 1 and 2, and 25 mg sulphadoxine per kg plus 1.25 mg pyrimethamine per kg on Day 0) was administered under supervision over the first three days. Visits for the follow-up were scheduled on Day 1, 2, 3, 7, 14, and 28. On every visit, patients were clinically examined and a Giemsa-stained blood slide was taken for the microscopic assessment of parasitaemia. A blood sample was taken on Day 0 (pre-treatment sample) and on Days 14 and 28 or any day of treatment failure for molecular genotyping purposes. At the end of the follow-up, the patients were classified according to their clinical and parasitological responses into early treatment failure (ETF), late clinical failure (LCF), late parasitological failure (LPF), or adequate clinical and parasitological response (ACPR) (WHO, 2003).

### Study sites and population

The studies were conducted between October 2003 and April 2004 in the Karimui area (Simbu Province) and the South Wosera area (East Sepik Province), two rural places mesoendemic for malaria but differing with regard to transmission intensity and drug use patterns [20]. Main characteristics of the study populations and the two sites are depicted in table 1.

**Table 1:** Baseline characteristics of study sites and patients at enrolment

	**Study site**	
**Characteristics**	**Karimui area **(Simbu Province)	**South Wosera **(East Sepik Province)
***Study sites***	n = 80	n = 94
Endemicity*	mesoendemic	mesoendemic
Transmission intensity^§^	moderate	high
***Patients***		

Weight (mean (95% CI), kg)	13.8 (12.9–14.6)	14.4 (13.8–15.1)
Age (mean (95% CI), yrs)	4.0 (3.7–4.4)	4.5 (4.2–4.8)
Sex: female/n (%)	43/97 (44.3)	59/112 (52.7)
Temperature (mean (95% CI),°C)	38.7 (38.5–38.9)	38.7 (38.4–39.0)
Haemoglobin (mean (95% CI), g/dl)	9.0 (8.6–9.5)	9.0 (8.7–9.3)
Parasite density (geometric mean (range), per μl)	21937 (1120–329400)	40526 (280–774400)
Multiplicity of infection (= MOI) (mean (95% CI))	1.48 (1.34–1.63)	1.73 (1.59–1.88)
Spleen rate^# ^(% (95% CI))	43.3 (33.3–53.7)	50.9 (41.3–60.5)

* Assessed by concomitant cross-sectional surveys in both study areas which showed *P. falciparum *prevalence rates of 11–50% in children aged 2–9 years (WHO, 2003); ^§ ^Müller *et al*., 2003; ^# ^proportion of children with enlarged spleen

Scientific approval and ethical clearance for the study was obtained from the Medical Research and Advisory Committee (MRAC) of the Ministry of Health in PNG and consent was obtained from parents or legal guardians prior to recruitment of each patient.

### Laboratory analyses

Fingerprick blood samples for molecular genotyping purposes were collected on Day 0 (pre-treatment sample) in EDTA microtainer tubes. DNA was extracted using QIAamp^® ^DNA Blood Kit (Qiagen, Hombrechtikon, Switzerland) according to the manufacturer's instructions.

Assessment of single nucleotide polymorphisms (SNPs) for drug resistant malaria was done for *pfmdr1 *codons N86Y, Y184F, S1034C, N1042D and D1246Y, *pfcrt *codons K76T, H97Q, T152A, S163R, A220S, Q271E, N326D/S, I356L/T and R371I, *pfdhfr *condons A16V, N51I, C59R, S108N/T and I164L, and *pfdhps *codons S436A, A437G, K540E, A581G, and A613T/S. The method is based on parallel PCR amplification of the target sequences followed by primer extension mediated mini-sequencing using fluorochrome-labelled ddNTPs. Subsequent base calling occurs on a microarray upon sequence specific hybridization [24].

Assessment of the multiplicity of infection (MOI) in pre-treatment samples and the differentiation between true recrudescences and new infections in treatment failure samples was done by PCR-RFLP analysis of the merozoite surface protein 2 (*msp2*) as previously described [25,26].

### Statistics

Statistical analyses were performed by the use of STATA software (version 8.2; Stata Corp., College Station, Texas). The strength of association was evaluated by calculating odds ratios (OR). χ^2 ^tests and Fisher's exact test and where applicable stepwise logistical regression analyses were used to assess the significance of association between known risk factors and single or multiple mutations and treatment failure.

To estimate the allele frequencies of resistance markers in the study sample set, a non-linear statistical model that takes into account the effects of varying multiplicity of infection and assumes that resistant and sensitive parasite clones are transmitted independently was applied. The likelihood of a sample containing no resistant clones is (1 - *p*)^*n*^, where *p *is the frequency for the mutant allele and *n *is the multiplicity of infection of the sample. Similarly, the likelihood for the sample to contain no wild-type allele is *p*^*n *^and for a mixture of both, a wild-type and a resistant allele, is 1 - *p*^*n *^– (1 - *p*)^*n*^. The likelihood over the whole data set for *p *is computed as the product of this likelihood over all samples, using values of *n *derived from *msp2 *genotyping results. Allele frequencies were added by maximising this likelihood using a simple one dimensional search routine. This gave very similar estimates to those made using a corresponding Bayesian algorithm [27]. Confidence intervals were calculated using bootstrap sampling.

## Results

### *In vivo *drug efficacy

A total of 80 patients in Karimui and 94 patients in the Wosera were enrolled into the study and treated with AQ+SP (median age of 4 years). Day-28 treatment failure rates for *P. falciparum *after PCR-correction, classifying infections with new and recurrent strains as true recrudescences (i.e., treatment failures), were 29% in the Karimui and 19% in the Wosera area, respectively (Table 2).

**Table 2:** Treatment outcomes for amodiaquine plus sulphadoxine-pyrimethamine against *P. falciparum *malaria in Papua New Guinea

	**Study sites**	
	**Karimui area**	**South Wosera area**
	n = 80	n = 94
Outcome*	Number (%)	

ACPR	57 (71.2)	74 (81.3)
ETF	1 (1.3)	5 (5.5)
LCF	6 (7.5)	1 (1.1)
LPF	16 (20.0)	11 (12.1)

Total TF	23 (28.8)	17 (18.7)

* PCR-corrected values up to Day 28; ACPR, Adequate clinical and parasitological response; ETF, Early treatment failure; LCF, Late clinical failure; LPF, Late parasitological failure; TF, Treatment failure

### Prevalence and relationship of *pfmdr1*, *pfcrt*, *pfdhfr *and *pfdhps *mutations

Mutation analyses were successfully accomplished in all 174 pre-treatment samples from both study sites. Polymorphisms were found in *pfmdr1 *codons N86Y, Y184F, and N1042D, *pfcrt *codons K76T, A220S, N326D and I356L, *pfdhfr *codons C59R and S108N, and *pfdhps *codons A437G and K540E. None of the other SNPs (11/24) was detected as mutated allele in any of the infections analysed.

**Figure 1:**
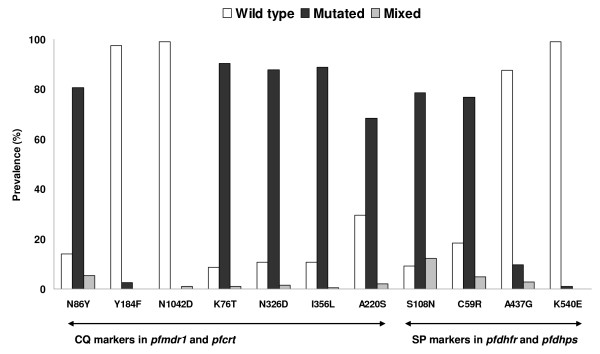
**Prevalence of mutations in *pfmdr1*, *pfcrt*, *pfdhfr *and *pfdhps *in patient samples from Papua New Guinea**. CQ, chloroquine; SP, sulphadoxine-pyrimethamine; *pfmdr1*, *Plasmodium falciparum *multidrug resistance gene 1; *pfcrt*, *Plasmodium falciparum *chloroquine resistance transporter; *pfdhfr*, *Plasmodium falciparum *dihydrofolate reductase; *pfdhps*, *Plasmodium falciparum *dihydropteroate synthase; no mutation was detected in any of the other SNP sites analysed (13/24 sites).

Regarding CQ relevant molecular markers, infections with mutated *pfmdr1 *N86Y and *pfcrt *K76T, N326D, I356L and A220S alleles were with 86%, 91%, 89%, 89%, and 70% very common, whereas 5%, 1%, 1%, 0%, and 2% of these infections were mixed with a wild-type allele (Figure 1). The mutated alleles in *pfmdr1 *Y184F and *pfmdr1 *N1042D were only found in five (3%) and two (1%) samples, respectively, with the latter being detected as mixed allele only. Considering relationships of mutated alleles in *pfcrt*, the mutations N326D and I356L were always linked, the double mutation N326D+I356L never occurred without a mutated allele K76T, and a mutation A220S never occurred without the triple mutation K76T+N326D+I356L. Considering *pfmdr1*, a mutation N1042D was always linked to a mutated Y184F allele, but these mutated alleles never occurred together with a N86Y mutation.

Regarding SP relevant molecular markers, mutations in *pfdhfr *S108N and C59R were also very common with 79% and 77% of infections having a pure mutant, and 91% and 82% of infections having a mutant or mixed allele, respectively. Mutated alleles in *pfdhps *A437G were found in 13% of all infections whereas in 10% it was detected as a pure mutant. The *pfdhps *K540E mutation was only found in two samples and was only detected as pure mutant allele. *Pfdhfr *C59R was never detected without *pfdhfr *S108N, and *pfdhps *mutations A437G and K540E were strongly linked to the double mutation *pfdhfr *S108N+C59R, with only two of the samples having the *pfdhps *A437G mutation with concomitant wild-type alleles in *pfdhfr*.

### Association between *pfmdr1*, *pfcrt*, *pfdhfr *and *pfdhps *alleles and treatment outcome

All patient isolates were coded according to presence or absence of mutant alleles and isolates showing both, wild-type and mutant allele, were treated as mutant. Likewise, infecting genotypes were coded according to the most highly mutated *pfmdr1*, *pfcrt*, *pfdhfr *and *pfdhps *alleles present in the sample.

Apart from *pfmdr1 *N86Y (OR = 7.87, 95% CI: 1.03–60.36, *p *< 0.01) and *pfdhps *A437G (OR = 3.44, 95% CI: 1.40–8.47, *p *< 0.01), there was no independent marker found to be significantly associated with treatment failure (Table 3). When known confounding factors, such initial parasite density, age, and multiplicity of infection (MOI), were adjusted for in a stepwise logistical regression model, the significant associations for the two above-mentioned markers were retained.

**Table 3:** Association between mutated single markers in *pfcrt*, *pfmdr1*, *pfdhfr *and *pfdhps *and treatment failure with amodiaquine plus sulphadoxine-pyrimethamine

**Gene**	**Polymorphism**	**OR**	**95% Confidence Interval**	***p *(LRT)**
***pfcrt***	K76T	2.09	0.45–9.70	0.31
***pfcrt***	I326L	2.64	0.58–12.03	0.16
***pfcrt***	N356D	2.64	0.58–12.03	0.16
***pfcr***	A220S	1.23	0.55–2.75	0.62

***pfmdr1***	N86Y	7.87	1.03–60.36	**<0.01**
***pfmdr1***	Y184F	^§^		
***pfmdr1***	N1042D	^§^		

***pfdhfr***	S108N	0.74	0.22–2.51	0.64
***pfdhfr***	C59R	2.34	0.77–7.14	0.11

***pfdhps***	A437G	3.44	1.40–8.47	**<0.01**
***pfdhps***	K540E	^§^		

OR, odds ratio; LRT, likelihood ratio test; *pfcrt*, *Plasmodium falciparum *chloroquine resistance transporter; *pfmdr1*, *Plasmodium falciparum *multidrug resistance gene 1; *pfdhfr*, *Plasmodium falciparum *dihydrofolate reductase; *pfdhps*, *Plasmodium falciparum *dihydropteroate synthase; ^§ ^mutated alleles were not detected in samples from treatment failure cases

Twenty three different genotypes could be discriminated with regard to mutated gene loci in all four genes analysed (Table 4). Among those, seven were observed in treatment failure cases, whereas the remaining 16 were exclusively found in patients with an adequate treatment response. Associations between these genotypes and treatment failure can be summarized as follows. Odds ratios for treatment failure were only increased for genotypes having the N86Y mutation in *pfmdr1 *combined with the double mutation S108N+C59R in *pfdhfr*, regardless of the concomitant genotype in *pfcrt*. However, the risk for failure reached only statistical significance for genotypes having the *pfmdr1 *N86Y mutation in conjunction with the quadruple mutation K76T+A220S+N326D+I356L in *pfcrt *and the A437G allele in *pfdhps *(OR = 3.84, 95% CI: 1.34–11.03, *p *< 0.01). Furthermore, a significant association was also observed with the genotype harbouring the *pfcrt *quadruple mutant plus *pfmdr1 *N86Y without any concurrent mutations in *pfdhfr *or *pfdhps *(OR = 7.17, 95% CI: 1.26–40.71, *p *= 0.02).

**Table 4:** Association between infecting *pfcrt*, *pfmdr1*, *pfdhfr *and *pfdhps *genotypes and treatment failure with amodiaquine plus sulphadoxine-pyrimethamine

**CQ-relevant markers**					**SP-relevant markers**							
***pfcrt***				pfmdr1*	***pfdhfr***		***pfdhps***					
K76T	N326D	I356L	A220S	N86Y	S108N	C59R	A437G	K540E	P (%)	OR	95% CI	*p *(χ^2^)

									1.2	^§^		
					**X**				0.6	^§^		
					**X**	**X**			1.7	^§^		
**X**									0.6	^§^		
**X**					**X**	**X**			0.6	^§^		
**X**	**X**	**X**			**X**	**X**			0.6	^§^		
**X**	**X**	**X**	**X**						0.6	^§^		
**X**	**X**	**X**	**X**		**X**				0.6	^§^		
**X**	**X**	**X**	**X**		**X**	**X**			**7.0**	0.28	0.03–2.24	0.16
				**X**					0.6	^§^		
				**X**	**X**				1.2	^§^		
				**X**	**X**	**X**			**3.5**	1.67	0.29–9.48	0.57
**X**				**X**	**X**	**X**			0.6	^§^		
**X**	**X**	**X**		**X**					0.6	^§^		
**X**	**X**	**X**		**X**	**X**				1.2	^§^		
**X**	**X**	**X**		**X**	**X**	**X**			**11.7**	1.10	0.38–3.25	0.86
**X**	**X**	**X**		**X**	**X**	**X**	**X**		**3.5**	3.46	0.67–17.86	0.15
**X**	**X**	**X**	**X**	**X**					**3.5**	7.17	1.26–40.71	**0.02**
**X**	**X**	**X**	**X**	**X**				**X**	1.2	^§^		
**X**	**X**	**X**	**X**	**X**	**X**				6.4	^§^		
**X**	**X**	**X**	**X**	**X**	**X**	**X**			**42.7**	1.00	0.48–2.03	0.98
**X**	**X**	**X**	**X**	**X**	**X**	**X**	**X**		**9.3**	3.84	1.34–11.03	**<0.01**
**X**	**X**	**X**	**X**	**X**	**X**	**X**		**X**	0.6	^§^		

* due to very low mutation rates, genotypes with mutated gene loci Y184F and N1042D in *pfmdr1 *were grouped together with the wild-type *pfmdr1 *genotypes; AQ, amodiaquine; SP, sulphadoxine-pyrimethamine; *pfcrt*, *Plasmodium falciparum *chloroquine resistance transporter; *pfmdr1*, *Plasmodium falciparum *multidrug resistance gene 1; *pfdhfr*, *Plasmodium falciparum *dihydrofolate reductase; *pfdhps*, *Plasmodium falciparum *dihydropteroate synthase; P, prevalence; OR, odds ratio; X, mutated allele; ^§ ^the genotype was not detected in samples from treatment failure cases

### Mutant allele frequencies and treatment outcome by site

**Figure 2:**
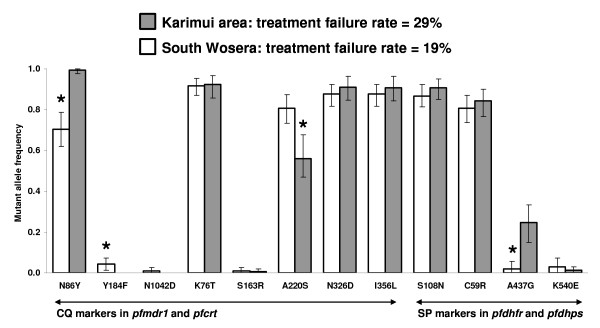
**Maximum likelihood estimates of mutant allele frequencies at the two study sites**. Error bars denote 95% confidence intervals; * denotes statistical significance at the 95% level; CQ, chloroquine; SP, sulphadoxine-pyrimethamine; *pfmdr1*, *Plasmodium falciparum *multidrug resistance gene 1; *pfcrt*, *Plasmodium falciparum *chloroquine resistance transporter; *pfdhfr*, *Plasmodium falciparum *dihydrofolate reductase; *pfdhps*, *Plasmodium falciparum *dihydropteroate synthase.

To investigate whether the difference in treatment outcome at the two study sites was reflected in the drug resistance profile of the corresponding parasites, mutant allele frequencies for each gene locus were calculated. Maximum likelihood estimates of mutant allele frequencies found in the two study populations are presented in Figure 2. Regarding the allele frequencies for the CQ relevant molecular markers, there was no significant difference in *pfcrt *K76T, N326D, and I356L. The only statistically significant differences in allele frequencies between the Karimui and the Wosera area were found for *pfcrt *A220S (0.56 versus 0.81, *p *< 0.0001), *pfmdr1 *N86Y (0.99 versus 0.70, *p *< 0.0001) and *pfmdr1 *Y184F (0.00 versus 0.04, *p *= 0.001). A similar picture was observed for the SP relevant molecular markers. Whereas the difference in any of the mutated loci in *pfdhfr *was not significant, the genetic profile for *pfdhps *mutation A437G was significantly different in the two parasite populations with an allele frequency of 0.25 in the Karimui area versus 0.02 in the Wosera area (*p *< 0.0001).

## Discussion

The genetic drug resistance profile was established in pre-treatment samples from malaria patients in Karimui and the Wosera by the use of a new DNA microarray-based technology [24] and its relationship with *in vivo *drug response to the combinations of AQ+SP was analysed. The principal objectives were to establish the baseline prevalence of polymorphisms in genes related to AQ/CQ and SP resistance, to assess their relationship with treatment outcome, in order to identify and propose useful markers for molecular monitoring of drug resistant *P. falciparum *in the country.

The analysis of the genetic profile of the parasite population revealed high levels of mutant alleles in CQ resistance (CQR) related *pfcrt *and *pfmdr1 *genes. The long history of 4-aminoquinoline use as monotherapy in PNG has led to a highly CQ resistant genetic background in the parasites as reported previously [21,28]. In addition, the results demonstrated prevalence rates of 91% and 82% for mutant alleles in the pyrimethamine related gene loci *pfdhfr *S108N and C59R. Mita *et al*. [29] recently analysed *P. falciparum *isolates from patients attending town clinics in Wewak (East Sepik Province) and observed similarly high prevalence rates of *pfdhfr *double S108N+C59R mutations (83% in 2002 and 86% in 2003). These high levels of mutation rates in *pfdhfr *appearing only a short time after the implementation of SP as one component of the official first-line policy were not surprising and may be due to i) the increasing recourse to SP as second-line therapy with quinine in the late 1990s (Nsanzabana *et al*., unpublished), ii) the former drug pressure exerted by the use of pyrimethamine (in combination with CQ) in mass drug administration campaigns in the 1960s and 1970s [30], and iii) the widespread use of trimethoprim-sulphamethoxazole for the treatment of bacterial infections [31,32]. Recent microsatellite analysis in *dhfr*-flanking regions by Mita *et al*. [29] revealed that the most prevalent *dhfr *haplotype (i.e., S108N+C59R double mutation) was associated with reduced microsatellite variability around the gene, an observation which argues for the selection of pre-existing SP resistant parasites, rather than the frequent emergence of *de novo *mutations in this gene [33]. These data further corroborate the hypothesis, that former drug pressure has lead to the emergence of pyrimethamine resistant parasites before the official introduction of SP in PNG.

Until 2003, polymorphic *pfdhps *loci associated with reduced sensitivity to sulpha drugs have only been found in a single *P. falciparum *isolate originating from PNG [21,22]. In the present study, prevalence rates of 13% for A437G and 1% for K540E were observed. Likewise, Mita and colleagues detected mutations in these loci in 8% of patient isolates collected in Wewak in the year 2003. In the view that *pfdhfr *mutations usually predominate over those in *pfdhp *[12,15], the detection of genotypes having a single *dhps *A437G mutation in combination with *pfdhfr *wild type alleles in two of the samples was rather unusual. However, this genotype may well have been selected by sulpha drugs used to treat infectious diseases other than malaria.

In order to propose a suitable marker set for the molecular monitoring of *P. falciparum *against the current combination therapy, the association of single mutations as well as infecting genotypes with *in vivo *treatment response was investigated. Regarding CQ relevant markers, the only single marker associated with a significantly increased risk of treatment failure was *pfmdr1 *N86Y. Taking into account additional SNPs in *pfcrt*, neither of the mutated alleles increased the predictive value for *pfmdr1 *N86Y, the most likely reason being that these mutations nearly reached fixed levels in the parasite population. Similarly, pyrimethamine relevant markers in *pfdhfr *did not show a significant association with treatment failure. Risk of failure was only increased with infections harbouring the A437G mutation in *pfdhps*. These observations are in agreement with previous studies showing that the prevalence of single molecular markers (e.g. *pfcrt *K76T or *pfdhfr *S108N) was almost always higher than the level of clinical or parasitological resistance to the respective drugs, especially in regions with high transmission intensity and long lasting drug pressure [4,34] and therefore, renders these markers unsuitable for molecular monitoring. Furthermore, the validity of molecular markers is dependent on former drug use and may also vary according to the malaria epidemiology in a given area [10,17,18]. The evaluation and assessment of a combination of markers, instead of single markers indicating the presence of a highly resistant genotype, have been suggested for the molecular monitoring of antimalarial resistance [12,14-16,35,36]. In the present study, which took into account the combined *pfcrt*/*pfmdr1*/*pfdhfr/pfdhps *genotype, the risk of treatment failure was clearly associated with the total number of mutations in the analysed genes. The risk was significantly increased for patients harbouring parasites with the most highly mutated genotype (i.e., 8/24 SNPs mutated). However, unusual findings included the increased risk of treatment failure with genotypes having the N86Y mutation in *pfmdr1 *and the quadruple mutation in *pfcrt *combined with a fully wild type *pfdhfr*+*pfdhps *allele. These results highlight again the fact that among many parasite and host factors, the molecular resistance background of *P. falciparum *is only one of several determinants for *in vivo *treatment outcome. Whereas acquired immunity can account for the clearance of drug resistant genotypes, diminished drug metabolism may well explain treatment failure in spite of an infection with a susceptible genotype [37].

Regarding former drug history in PNG, the relevance of key *pfdhps *mutations in predicting treatment failure was expected. AQ and CQ as inefficacious partner drugs of SP in the new standard regimen were not able to curb both, the progression of pyrimethamine resistance as well as the emergence of sulphadoxine resistance. It is most likely that in this sample, clinical efficacy of the sulpha component was mainly assessed. However, according to the present results, also *pfmdr1 *N86Y plays an important role in predicting a negative treatment response. CQ and AQ are chemically related drugs and cross-resistance has been described in several clinical and *in vitro *reports. Though little is known about the genetic mechanisms conferring AQ resistance [38], an important role has been ascribed to the key CQR markers *pfcrt *K76T and *pfmdr1 *N86Y [39,40]. It has been shown recently that in combination with *pfcrt *K76T, the *pfmdr1 *N86Y polymorphism was predictive for treatment failure with AQ in Nigeria [41] and that AQ resistance was associated with the selection of these polymorphisms in Kenya [42]. Considering the long use of AQ as monotherapy against uncomplicated falciparum malaria in PNG and the observation that *pfmdr1 *N86Y is a strong predictor for treatment failure with AQ+SP, the present data support the hypothesis that *pfmdr1 *N86Y is probably involved in AQ resistance. Several studies have shown that both, SNPs and gene amplification of *pfmdr1*, can mediate resistance to 4-aminoquinlines and also other drug classes, such as amino alcohols and artemisinin derivates [43] However, results from different studies investigating the relationship of these genetic alterations in *pfmdr1 *and *in vivo *response were often inconsistent [6,44]. Direct (active drug translocation) and indirect (modification of biophysical cell parameters) modes of action have been proposed for P-glycoprotein homolog 1, the gene product of *pfmdr1*. But how genetic alterations in *pfmdr1 *and epistatic interactions with other genes finally lead to a multidrug resistant phenotype remains to be resolved [45].

Finally, the fact that the difference in clinical outcome between the two sites was reflected in the genetic profile of the corresponding parasite populations, especially for the frequencies of *pfmdr1 *N86Y and *pfdhps *A437G, further confirmed the role of these two markers as important predictors for a negative treatment response with AQ+SP and suggests them to be the most useful resistance surveillance markers with the current standard treatment in PNG.

## Conclusion

This study shows that a careful baseline assessment of molecular markers, including the investigation of their relationship with treatment response, is essential for the identification of appropriate marker sets. For the parallel analysis of SNPs in multiple genes in a large sample size, DNA microarray technology has proven to be a valuable and cost-effective tool. However, the use of additional markers could become necessary for the longitudinal resistance monitoring in the future, in particular when current drug policy starts to show reduced effectiveness. These may include SNPs in known or as yet uncharacterized genes involved in resistance to the commonly used antimalarials, or markers against newly implemented drug classes, such as the artemisinins.

## Authors' contributions

JM participated in the coordination of the field and laboratory studies, performed data acquisition and molecular and statistical analyses and drafted the manuscript. IM, AS, OO and JCR participated in the coordination of the field studies. BG, HPB, and TAS participated in the design of the study, the statistical analysis and the drafting of the manuscript. All authors read and approved the final manuscript.
